# Survey of clinical practice pattern in Germany’s certified chest pain units: Adherence to the European Society of Cardiology guidelines on non-ST-segment elevation acute coronary syndrome

**DOI:** 10.1007/s00059-021-05079-2

**Published:** 2021-11-09

**Authors:** Frank Breuckmann, Stephan Settelmeier, Tienush Rassaf, Felix Post, Winfried Haerer, Johann Bauersachs, Harald Mudra, Thomas Voigtländer, Jochen Senges, Thomas Münzel, Evangelos Giannitsis

**Affiliations:** 1 Department of Cardiology and Vascular Medicine, West German Heart and Vascular Center Essen, University Duisburg-Essen, Essen, Germany; 2 Department of Cardiology, Katholisches Klinikum Koblenz-Montabaur, Koblenz, Germany; 3 Heart Clinic Ulm, Ulm, Germany; 4 Department of Cardiology and Angiology, Hannover Medical School, Hannover, Germany; 5 Heart and Vascular Center Munich Maffeistraße and Nymphenburg (Klinikum 3. Orden), Munich, Germany; 6 CCB, Cardioangiologisches Centrum Bethanien, Frankfurt am Main, Germany; 7 Institute for Myocardial Infarction Research Foundation, Ludwigshafen, Germany; 8 Department of Cardiology, University Medical Center Mainz, Johannes Gutenberg-University Mainz, Mainz, Germany; 9 Department of Medicine III, University Hospital Heidelberg, Heidelberg, Germany

**Keywords:** Standard of care, Timing, Troponin protocol, PCI, Antiplatelet therapy, Versorgungsrealität, Zeitplanung, Troponin-Protokoll, Perkutane koronare Intervention, Plättchenhemmungstherapie

## Abstract

**Background:**

We aimed to analyze the 2020 standard of care in certified German chest pain units (CPU) with a special focus on non-ST-segment elevation acute coronary syndrome (NSTE-ACS) through a voluntary survey obtained from all certified units, using a prespecified questionnaire.

**Methods:**

The assessment included the collection of information on diagnostic protocols, risk assessment, management and treatment strategies in suspected NSTE-ACS, the timing of invasive therapy in non-ST-segment elevation myocardial infarction (NSTEMI), and the choice of antiplatelet therapy.

**Results:**

The response rate was 75%. Among all CPUs, 77% are currently using the European Society of Cardiology (ESC) 0/3‑h high-sensitive troponin protocol, and only 20% use the ESC 0/1‑h high-sensitive troponin protocol as a default strategy. Conventional ergometry is still the commonly performed stress test with a utilization rate of 47%. Among NSTEMI patients, coronary angiography is planned within 24 h in 96% of all CPUs, irrespective of the day of the week. Prasugrel is the P2Y12 inhibitor of choice in ST-segment elevation myocardial infarction (STEMI), but despite the impact of the ISAR-REACT 5 trial on selection of antiplatelet therapy, ticagrelor is still favored over prasugrel in NSTE-ACS. If triple therapy is used in NSTE-ACS with atrial fibrillation, it is maintained up to 4 weeks in 51% of these patients.

**Conclusion:**

This survey provides evidence that Germany’s certified CPUs ensure a high level of guideline adherence and quality of care. The survey also identified areas in need of improvement such as the high utilization rate of stress electrocardiogram (ECG).

## Abstract (de)

**Hintergrund:**

Ziel der Arbeit war eine Versorgungsanalyse für das Jahr 2020 im Hinblick auf das akute Koronarsyndrom ohne ST-Strecken-Hebung (NSTE-ACS), welche anhand eines standardisierten Fragebogens in allen zertifizierten Brustschmerzeinheiten (CPU) in Deutschland durchgeführt wurde.

**Methoden:**

In die Bewertung flossen Diagnoseprotokolle, Risikobewertung, Management- und Behandlungsstrategien bei Verdacht auf NSTE-ACS, Zeitpunkt der invasiven Therapie beim Nicht-ST-Strecken-Hebungs-Myokardinfarkt (NSTEMI) und die Wahl der Thrombozytenaggregationshemmung ein.

**Ergebnisse:**

Die Rücklaufquote betrug 75 %. Von allen CPU verwenden derzeit 77 % das ESC 0/3-h-high-sensitive-Troponin-Protokoll, und nur 20 % haben das ESC 0/1-h-high-sensitive-Troponin-Protokoll als Standardstrategie. Die konventionelle Ergometrie ist mit einer Durchführungsrate von 47 % nach wie vor der am häufigsten durchgeführte Belastungstest. Bei NSTEMI-Patienten wird in 96 % aller CPU, unabhängig vom Wochentag, eine Koronarangiographie innerhalb von 24 h geplant. Prasugrel ist der P2Y12-Inhibitor der Wahl beim ST-Strecken-Hebungs-Myokardinfarkt. Trotz der Ergebnisse der ISAR-REACT-5-Studie wird Ticagrelor beim NSTE-ACS immer noch gegenüber Prasugrel bevorzugt. Wird eine Tripeltherapie beim NSTE-ACS mit Vorhofflimmern eingeleitet, wird diese in 51 % der CPU bis zu 4 Wochen empfohlen.

**Schlussfolgerung:**

Die vorliegende repräsentative Umfrage unter den zertifizierten CPU liefert Belege für ein hohes Maß an Leitlinienkonformität und Versorgungsqualität in diesen Einheiten. Die Analyse deckt jedoch auch Bereiche mit Verbesserungsbedarf (wie die unverändert hohe Rate an Belastungs-EKG) auf.

By the end of the year 2020, 292 certified chest pain units (CPUs) were established to form a network across Germany with almost complete nationwide coverage [1, 2]. The certification process was organized under the umbrella of the German Cardiac Society (Deutsche Gesellschaft für Kardiologie [DGK]; [3]). Dedicated certification criteria with periodic updates have been developed and are being constantly adapted to comply with the European Society of Cardiology (ESC) guidelines. Successful certification requires minimum characteristics on location, equipment, diagnostic and therapeutic strategies, collaboration with a cardiac surgery, cardiology outpatient services, and continuous staff education [4–6]. The CPU initiative was accompanied by voluntary participation in a German CPU registry that provided valuable information on performance and outcome measures. Unfortunately, inclusion in the German CPU registry was recently stopped and a central national registry for quality assessment and benchmarking such as the Swedish SWEDEHEART registry is no longer available [7, 8]. Thus, there is a lack of objective data concerning the current standard of care and guideline adherence across the certified units. Guideline adherence has been identified to represent a predictor of outcome [9]. Therefore, the present survey focused on indicators of guideline adherence and clinical practice in suspected or confirmed acute coronary syndrome (ACS). The items of the survey included questions about the diagnostic strategy, acute antiplatelet treatment, timing of invasive management, and duration of triple therapy in patients with non-ST-segment elevation acute coronary syndrome (NSTE-ACS) and atrial fibrillation (AF) undergoing percutaneous coronary intervention (PCI).

Recently, the diagnosis and management of patients with NSTE-ACS was updated in the 2020 ESC guidelines that were published online after the completion of the present survey [10]. Therefore, we now aimed to analyze the 2020 standard of care in certified German CPUs and compare these current standards with the recommendations of the 2015 and the 2020 ESC guidelines [11]. The survey was conducted on a voluntary basis hiding identifying information of the participating CPU or the lead physician.

## Methods

Certified units were identified via the official website of the DGK [1]. The CPUs were officially invited by the DGK for voluntary participation to answer a standardized questionnaire for regular quality surveillance. The survey was reviewed by the institutional review board of the DGK and formal ethics approval was waived. Surveys were sent out to all certified CPUs across Germany. The questionnaire contained 15 questions covering five topics as highlighted below. Following formal consent to participate, CPUs had the choice to reply in written form or by telephone interview. In the case of any questions or multiple answers for individual questions when the written form was preferred, a subsequent telephone interview was conducted for clarification. The interview was carried out either by interviewing the head of the department or the head of the CPU at the discretion of each CPU. All data were anonymized for the participating CPU. Interviews were performed over a period of 8 weeks. The due date was August 1, 2020.

### Questionnaire and evaluation points

A total of 15 evaluation benchmark questions were composed in a multiple-choice manner with a variable number of answers (Supplementary Information). The answer best describing the local practice should be selected for each question. The participants were asked to answer intuitively. The following categories were addressed:

#### CPU characterization and basic demographics

Identification of location and assignment to a federal state was based on the ZIP code. The federal states Schleswig-Holstein, Mecklenburg-West Pomerania, Lower Saxony, Hamburg, and Bremen were allocated as *north*, Berlin, Brandenburg, Saxony-Anhalt, North Rhine-Westphalia, Saxony, and Thuringia as *central,* and Rhineland-Palatinate, Hesse, Baden-Wuerttemberg, Saarland, and Bavaria as *south*. The type of hospital distinguished between (a) university hospital, (b) academic teaching hospital, and (c) other health facility providers such as primary care in a community hospital, as described elsewhere [2, 12]. The estimated number of patients per day was asked and categorized semiquantitatively as < 5, 5–10, and > 10. Likewise, the anticipated percentage of self-referrals was estimated as < 25%, 25–50%, and > 50%. The anticipated numbers of percutaneous coronary interventions for CPU patients per year were also recorded in the following categories: < 250, 250–500, 500–1000, and > 1000 cases.

#### High-sensitive cardiac troponin protocol and score assessment

The questions included information on the local high-sensitive cardiac troponin (hs-cTn) assay and the diagnostic protocol. Options for reply included the ESC guideline recommended protocols, i.e., the ESC 0/1‑h protocol, the ESC 0/3‑h protocol if a hs-cTn assay was available, or the 0‑h/6–12‑h retesting protocol that is only recommended when hs-cTn assays are not available. In addition, the CPUs were asked about the regular use of scoring systems to determine the ischemic as well as the bleeding risk.

#### Diagnostic approach in troponin-negative NSTE-ACS

Participants were asked to choose the preferred next diagnostic step in their CPU after ruling out acute myocardial infarction (MI). Choices included coronary angiography within 72 h irrespective of further risk stratification, coronary angiography in patients with secondary risk markers, or a selective invasive strategy after cardiac computed tomography, stress testing, or clinical judgement. If stress testing was chosen as the diagnostic step of first choice, the CPUs were asked to differentiate between stress ECG, stress echocardiography, stress magnetic resonance imaging, or myocardial scintigraphy.

#### Timing of invasive therapy in troponin-positive NSTE-ACS

For patients with non-ST-segment elevation MI (NSTEMI) at very high risk, participants were asked about the rate of using immediate coronary angiography (analogous to STEMI) or the rates of using an early invasive strategy. For patients with NSTEMI but without criteria indicating a very high risk, participants were asked to name their CPU’s usual timing of invasive diagnostics, thereby distinguishing between coronary angiography within 2 h, within 2–12 h (or on the same day of admission), within 12–24 h (or on the next day), or within 72 h. Additionally, the preferred strategy for NSTEMI patients was enquired for patients presenting on Fridays after routine working hours or during the weekend. Participants were given the choice between coronary angiography < 12 h (or on the same day), within 12–24 h (or on the next day) or during regular working hours on Monday mornings.

#### Choice of antiplatelet therapy with and without AF

Participants were asked to name their CPU’s preferred antiplatelet agent (having the choice between clopidogrel, prasugrel, or ticagrelor) in combination with acetylsalicylic acid (ASA), separately for patients with STEMI, NSTEMI, and unstable angina undergoing PCI. Additionally, the survey asked whether the findings of the ISAR-REACT 5 trial published in 2019 had already influenced their choice of P2Y12 inhibitor [13].

For patients with NSTE-ACS and AF, the duration of triple therapy was recorded. Possible choices comprised duration of dual antiplatelet therapies during hospital stay for 1 week, 1–4 weeks, 4 weeks to 3 months, more than 3 months, or no triple therapy at any time. Regardless of the duration of triple therapy, CPUs were asked to provide details on the P2Y12 inhibitor used, i.e., use of clopidogrel only, mainly clopidogrel with alternative use of ticagrelor in individual cases, or mainly clopidogrel with alternative use of prasugrel in individual cases. Further, CPUs were asked for the preferred type of anticoagulation, i.e., administration of vitamin K antagonists (VKA) or new oral anticoagulants (NOAC). If NOACs were preferred, specification of the dose, i.e., standard dose versus reduced dose, was required.

### Statistical analysis

All data are provided in a descriptive approach without further statistical analysis.

## Results

The response rate to the interview was 75%, allowing data collection from 214 of the 287 certified units by August 1, 2020.

### CPU characterization and basic demographics

A total of 35 university hospitals, 148 academic teaching hospitals, and 31 community hospitals responded to the questionnaire. A total of 52 CPUs were allocated to northern Germany, 132 to central Germany, and 105 to southern Germany. At nearly equal levels, CPUs reported mean numbers of admittance of < 5 (42%) or 5–10 (44%) chest pain patients per day. Self-admission was estimated at < 25% in 28%, 25–50% in 60%, and > 50% in 12% without relevant differences between the different regions. University hospitals reported highest rates for the 5–10 group of CPU admissions, whereas community hospitals peaked at < 5. More than half of the CPUs (56%) and 26% of CPUs reported annual PCI rates of 250–500 or 500–1000, respectively. University hospitals reported the highest number, followed by academic teaching hospitals and community hospitals (Table 1).

**Table 1:** Chest pain unit (CPU) characterization and basic demographics depending on hospital type and geographical allocation

	CPU admissions per day			Self-admission			Yearly PCI rates			
	< 5 (%)	5–10 (%)	> 10 (%)	< 25% (%)	25–50% (%)	> 50% (%)	< 250 (%)	250–500 (%)	500–1000 (%)	> 1000 (%)
**Total**										
*Total*	42	44	14	28	60	12	14	56	26	5
North	38	47	15	32	56	12	18	62	18	3
Central	41	47	12	24	59	17	10	57	29	4
South	46	39	15	31	64	5	16	51	26	6
**University hospitals**										
*Total*	3	9	5	5	9	3	1	5	8	3
North	6	6	3	6	9	0	0	9	6	0
Central	5	10	3	5	8	5	2	7	9	0
South	0	8	8	4	11	0	0	1	8	6
**Academic teaching hospitals**										
*Total*	30	32	7	17	43	9	9	42	16	2
North	26	32	9	18	38	12	12	44	9	3
Central	28	35	7	15	44	11	6	43	17	4
South	35	29	5	19	45	5	11	40	18	0
**Other health facilities**										
*Total*	9	3	3	7	7	0	4	8	2	0
North	6	9	3	9	9	0	6	9	3	0
Central	8	2	2	2	4	7	1	2	7	3
South	11	3	3	9	8	0	5	10	1	0

*CPU* chest pain unit, *PCI* percutaneous coronary interventions

### hs-cTn protocol and score assessment

**Fig. 1:**
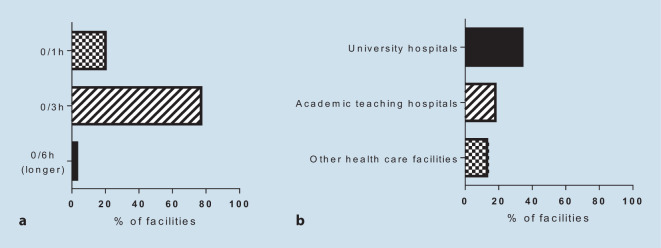
Different use of high-sensitive troponin protocols in certified German chest pain units in total (**a**) and in a subanalysis for the 0/1‑h protocol by facility type (**b**)

The ESC 0/1‑h protocol was reported as the most commonly employed troponin protocol in 20% of CPUs involved in this survey, whereas the ESC 0/3‑h protocol was chosen as the strategy of choice in the majority of CPUs (77%). Longer protocols were stated to be favored by 3% only. The highest proportions of the ESC 0/1‑h protocol were from university hospitals (34%, Fig. 1a,b). Among all CPUs, 63% reported use of a clinical score to assess the ischemic risk, whereas 42% reported use of a score to assess the risk of bleeding, both without relevant differences between the levels of healthcare providers.

### Diagnostic approach in unstable angina or after ruling out MI

**Fig. 2:**
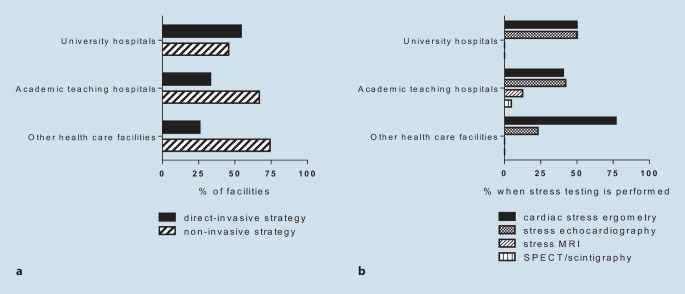
Direct-invasive vs. primarily noninvasive diagnostic approaches (**a**) and stress testing of choice (**b**) in troponin-negative non-ST-segment elevation acute coronary syndrome

Most CPUs (39%) responded to the use of objective stress test criteria to select between an invasive or conservative strategy. Among all CPUs, 21% answered that their decision is mainly based on the absence or presence of secondary risk criteria for early invasive management. Use of cardiac computed tomography was reported in 5%. Subjective clinical decision or coronary angiography within 72 h was reported by 21% or 14%, respectively. Whereas academic teaching hospitals and community hospitals seemingly favored a noninvasive approach, university hospitals more often chose invasive management (Fig. 2a). When the answer “stress testing” was chosen, both university hospitals as well as academic teaching hospitals preferred conventional ergometry or stress echocardiography at nearly equal levels, community hospitals reported conventional ergometry as the stress test of choice (77%, Fig. 2b).

### Timing of invasive therapy in troponin-positive NSTE-ACS

**Fig. 3:**
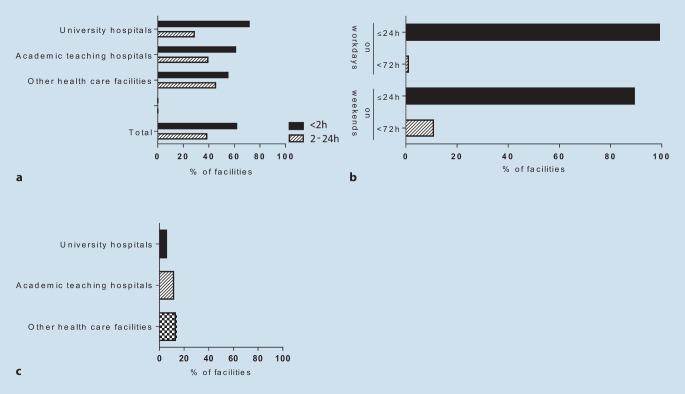
Timing of invasive therapy in patients with non-ST-segment elevation acute coronary syndrome (NSTE-ACS) at very high risk (**a**) or in troponin-positive NSTE-ACS patients without criteria of urgent invasive management (**b**); 11% of CPUs stated that invasive diagnostics are often postponed to the next Monday (**c**)

In NSTE-ACS patients with criteria of very high risk, 62% CPUs reported scheduling an invasive strategy analogous to STEMI patients with coronary angiography at the earliest occasion. Hospitals in southern and central Germany as well as university hospitals and academic teaching hospitals reported the highest percentages of immediate invasive management (Fig. 3a). As much as 99% of CPUs in this survey reported scheduling coronary angiography in NSTEMI within the first 24 h of admission without local or hierarchical differences. In 89%, this planned strategy was also followed on weekends and only 11% of the CPUs would postpone an invasive strategy to Mondays (Fig. 3b). Of those, university hospitals showed the highest rate of guideline adherence (Fig. 3c).

### Choice of antiplatelet therapy in ACS patients without AF

**Fig. 4:**
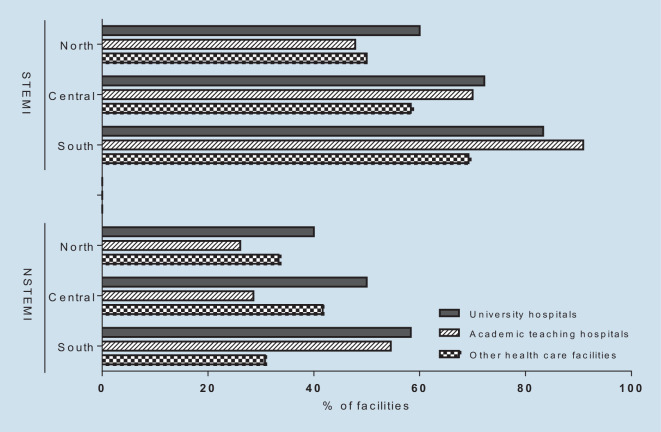
Prasugrel administration in ST-segment elevation myocardial infarction (*STEMI*) and non-ST-segment elevation myocardial infarction (*NSTEMI*) patients dependent on hospital type and geographical allocation

Detailed data on the preferential choice of the different P2Y12 inhibitors across the spectrum of ACS manifestations are given in Table 2. Prasugrel was selected most frequently for STEMI patients (72%), whereas ticagrelor was more often preferred for NSTEMI patients (58%), and clopidogrel (45%) or ticagrelor (38%) for patients with unstable angina undergoing PCI. In NSTEMI, only university hospitals tended to prefer administration of prasugrel. In addition, there was a regional preference in favor of prasugrel in the south as opposed to ticagrelor in the north (Fig. 4). Among all CPUs, 54% declared that the ISAR-REACT 5 results somehow changed their prescription behaviors.

**Table 2:** Choice of antiplatelet therapy in acute coronary syndrome (ACS) patients without atrial fibrillation

	STEMI			NSTEMI			Troponin-negative ACS		
	Prasugrel (%)	Ticagrelor (%)	Clopidogrel (%)	Prasugrel (%)	Ticagrelor (%)	Clopidogrel (%)	Prasugrel (%)	Ticagrelor (%)	Clopidogrel (%)
**Total**									
*Total*	72	28	0	40	58	2	17	38	45
North	50	50	0	29	68	3	18	41	41
Central	69	31	0	34	65	1	14	42	44
South	86	14	0	51	46	3	21	33	46
**University hospitals**									
*Total*	74	26	0	51	49	0	17	43	40
North	60	40	0	40	60	0	20	20	40
Central	72	28	0	50	50	0	11	44	44
South	83	17	0	58	42	0	25	42	33
**Academic teaching hospitals**									
*Total*	74	26	0	38	60	2	16	40	44
North	48	52	0	26	70	4	13	43	43
Central	70	30	0	29	70	1	13	43	44
South	91	9	0	55	44	2	22	35	44
**Other health facilities**									
*Total*	61	39	0	35	61	3	23	26	52
North	50	50	0	33	66	0	33	33	33
Central	58	42	0	42	58	0	25	33	42
South	69	31	0	51	46	3	21	33	46

*STEMI* ST-segment elevation myocardial infarction, *NSTEMI* non-ST-segment elevation myocardial infarction

### Choice of antiplatelet therapy in ACS patients with AF

**Fig. 5:**
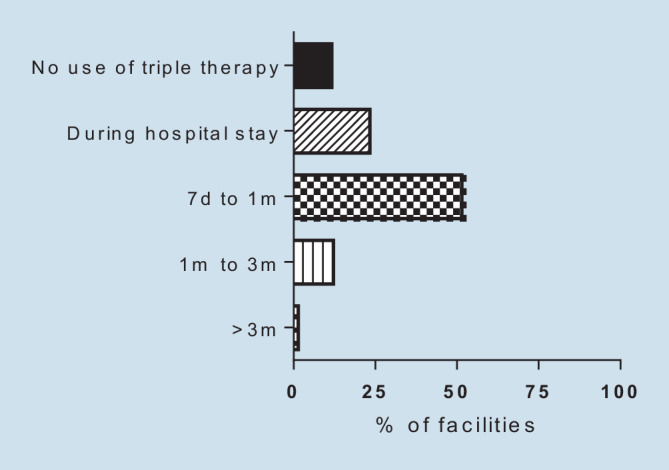
Default set of duration of triple therapy in patients with acute coronary syndrome and atrial fibrillation (*m* months, *d* days)

Triple therapy was selected by 88% of the CPUs of this survey. About half of the CPUs (51%) stated a duration of 1 week to 1 month as default strategy, 23% chose a reduced duration to the length of the hospital stay, whereas 14% favored a prolonged administration of more than 1 month (Fig. 5). No relevant differences were observed among the different levels of healthcare providers. Nearly all CPUs favored NOAC administration (99%) over VKA during triple therapy. A preferred administration of a reduced dosage of NOAC was reported from 39% of the CPUs. University hospitals reported the highest preference of full NOAC dosage as their default strategy, at 77%. Whereas 73% of CPUs chose clopidogrel for combination with aspirin in triple or dual therapy in NSTE-ACS with an indication for oral anticoagulation, 24% of CPUs stated sometimes switching to ticagrelor, which was similar among the levels of healthcare providers.

## Discussion

The process for CPU certification in Germany is regulated by a certification criteria consensus document that is periodically updated by the Task Force for CPU certification on behalf of the DGK; more than half of all hospitals running a cath lab have a certified CPU [3–5, 14]. The latest update was released mid-2020, shortly before the online publication of the ESC guidelines on NSTE-ACS during the annual conference of the ESC [5]. Thus, the CPU certification criteria did not adopt 2020 ESC guideline recommendations but were independent and based on recommendations from the 2015 ESC guidelines on NSTE-ACS as well as on evidence that was published thereafter [5, 10, 11]. The 2020 ESC guidelines made significant changes compared to the previous 2015 version [10].

### Diagnostic strategy

Accumulating evidence from several observational trials, a randomized controlled trial, and a meta-analysis supports the diagnostic accuracy and safety of the ESC 0/1‑h algorithm [15–20]. Although the ESC 0/3‑h algorithm that is based on the 99th percentile upper limit of normal has been established for years, direct comparisons of the ESC 0/1‑h versus the ESC 0/3‑h algorithm demonstrate small benefits in favor of the ESC 0/1‑h algorithm that are largely restricted to the numbers of patients qualifying for rule-out [21]. Therefore, a class IIa recommendation in favor of the ESC 0/1‑h algorithm is not unequivocally supported by robust evidence. Moreover, the evidence supporting the ESC 0/2‑h algorithm as an alternative to the ESC 0/1‑h algorithm, and thus in preference of the ESC 0/3‑h strategy, is not unopposed. The recommendation is limited by the fact that the ESC 0/2‑h algorithm is not widely distributed, and that this strategy has been almost completely derived from the APACE registry, with subsequent external validation against the accelerated diagnostic protocol of the 0/2‑h algorithm tested in the ADAPT study cohort [16, 22]. In their recent updated version of the certification criteria, the DGK also recommended a 0/1‑h or 0/2‑h protocol over a 0/3‑h protocol whenever a validated test is available [5]. According to our data, to date, German certified CPUs still perform a 0/3‑h protocol. This affects primarily non-university hospitals, but also less than a half of university hospitals are currently using a 0/1‑h protocol. Therefore, broad adaptions will be necessary to translate the ESC recommendations to the specific local setting as we anticipate that most of the certified units already have a test that is suitable for a faster diagnostic approach. Besides its accuracy in ruling-in ACS patients to NSTEMI patients, it will enable decision-making and ruling-out MI 2 h earlier than the 0/3‑h protocol, thereby ensuring a faster workflow in the CPU and a shorter time until direct discharge of low-risk individuals. Keeping in mind that most CPUs in Germany are located within an emergency room setting, this advantage may be of additional relevance during the current COVID-19 pandemic [12].

### Risk assessment and invasive management—low risk

The 2020 ESC guidelines propose a very different algorithm for the timing of the invasive strategy from the former 2015 ESC recommendations as the group of patients with intermediate-risk indicators assigned to a delayed invasive strategy is now downgraded in risk and is shifted to a selective invasive strategy. The decision for invasive angiography should be based on stress testing, preferably stress imaging and computed coronary angiography [10, 11]. Even if not strictly performed, at present, the certification update of the DGK still recommends a delayed invasive strategy for patients at intermediate risk whereas a selective invasive strategy is still only recommended for patients with unstable angina without additional risk indicators [5]. A debate has started on whether the 2020 recommendations of the ESC are supported by appropriate evidence. Regarding the latter, a recent meta-analysis on the benefits of earlier timing of invasive management did not demonstrate significant survival benefits in the overall pooled analysis but identified subgroups of patients with mortality benefits from an earlier invasive management including patients > 75 years, patients with a GRACE score > 140, those with diabetes mellitus, and patients with elevated cardiac troponin concentrations (not necessarily with a rise and/or fall of serial concentrations; [23]). However, these patients were not shifted to the early invasive but to the selective invasive group. The issue that emerged with the shift from invasive to noninvasive imaging is the anticipated increase in the number of stress imaging and/or coronary computed tomography examinations. Unfortunately, the survey disclosed a severe underutilization of stress imaging. In line with previous data from the German CPU registry, the proportion of cardiac computed tomography in unstable angina patients remains low at about 5% [8]. Interestingly, more than half of the CPUs still rely on conventional stress ergometry, although its use for diagnostic purposes was already discouraged in the 2019 ECS guidelines on chronic coronary syndrome due to inappropriate performance, and more specific tests are favored in the latest CPU certification criteria [5, 24, 25]. We anticipate that this might still be due to local barriers to more contemporary stress tests such as stress echocardiography, stress magnetic resonance tomography, or myocardial scintigraphy resulting from reduced local experience in some nonacademic hospitals, cutback in local resources for timely performance (both personnel and/or equipment), or a lack of adequate reimbursement for time- and resource-consuming alternatives. Whether or not a primarily noninvasive approach independent of the presence of secondary risk markers will also be advised to the German CPUs remains a matter of discussion and will depend on the national commentary on the ESC guidelines by the DGK.

### Risk assessment and invasive management—high to very high risk

Risk criteria for NSTE-ACS patients at very high risk as well as at high risk remained broadly unchanged. Still, criteria of very high risk should trigger immediate invasive measures just as in STEMI patients, whereas high-risk criteria should trigger early coronary angiography within 24 h [10]. For the latter category, while regular ischemic risk assessment at admission (e.g., GRACE-scoring) was weak at about 60%, our current survey demonstrates a nearly optimal in-hospital care as far as the timing of coronary angiography in NSTEMI patients is concerned. The CPU physicians indicated an optimistic expectation that about 99% of all NSTE-ACS patients at high risk are scheduled for coronary angiography within 24 h. Almost all CPUs state that early invasive strategy can be provided on weekdays and weekends, supporting previous data from the German CPU registry on on- and off-hour care in STEMI patients and single-center experience in NSTEMI patients [8, 26]. However, in 2018, CPU registry data controversially reported on a treatment paradox regarding the timing of early PCI in NSTEMI patients at very high risk. Overall, the proportion of patients who underwent coronary angiography within 24 h was almost 80% for patients with a NSTEMI diagnosis [27]. Unexpectedly, delays in coronary angiography were substantially longer as patients’ risk increased [28]. In our current survey, those patients at very high risk were identified with an indication for immediate coronary angiography within 2 h, or analogous to STEMI patients, in 62% of all CPUs. The higher the level of care, the higher the proportion of timely coronary angiography, which was best among university hospitals at about 80%. Interestingly, there was also a higher guideline adherence in southern and centrally located hospitals. This might be explained by the former-documented better CPU network coverage in southern and central/western parts of Germany [2].

### Selection of antiplatelet therapy in patients undergoing PCI, and deferred routine pre-treatment with P2Y12 inhibitors in patients planned for early PCI

According to the 2020 ESC guidelines, clopidogrel should be used when ticagrelor or prasugrel are not available, or when the novel antiplatelets are contraindicated (class IC). The selection the P2Y12 and the duration of a dual therapy should be based on the ischemic/bleeding risk balance of each patient. In agreement with precedent guidelines, prasugrel and ticagrelor are recommended with a class IB recommendation [10]. A new recommendation to prefer prasugrel over ticagrelor in patients planned for PCI (class IIa) is based on the subgroup of patients with NSTE-ACS from the ISAR-REACT 5 trial [13]. It is interesting to observe that the ISAR-REACT 5 trial already influenced the selection of antiplatelets, even before the online publication of the ESC guidelines. The current survey reveals that CPUs prefer the administration of prasugrel for STEMI whereas ticagrelor still remains the P2Y12 inhibitor of first choice for NSTEMI patients. The regional preference of prasugrel over ticagrelor in the southern parts of Germany at university and academic teaching hospitals but not at other healthcare facilities is difficult to interpret. It might be related to a selective multiplicator function of individual key opinion leaders or the effect of focused symposia.

### Duration of triple therapy in patients with AF and NSTE-ACS undergoing PCI

According to the ESC guidelines, NOACs should be preferred over VKA in the absence of contraindications whenever patients with NSTE-ACS and nonvalvular AF receive PCI [10, 29]. The ESC guidelines recommend a default setting with duration of triple therapy for up to 1 week, and a switch to dual therapy with a NOAC plus clopidogrel for up to 12 months. Our survey hereby demonstrates that those studies are already integrated in the daily practice of CPUs, as nearly all CPUs are using NOACs and restricting triple therapy but 75% of CPUs prefer to administer a dual antiplatelet therapy for a maximum of 4 weeks. Interestingly, about 25% of CPUs are also using ticagrelor in combination with NOAC adjusted, a strategy that lacks broad evidence from randomized controlled trials and is not recommended by 2020 ESC guidelines [30–33].

### Additional data originating from the survey

Estimations of the representative CPU delegates of our study suggest that nearly three quarter of all CPU patients are self-referrals, a proportion that is higher than previous data originating from the German CPU registry [8]. During the last few years and with increasing awareness, we anticipate a shift toward a lower threshold to self-refer to a certified CPU. Even though early self-referral is preferred over late activation of emergency medical service, oncoming CPU improvement efforts should address an increasing “open CPU concept,” redirecting patient flow and ensuring early risk assessment thereby underscoring the importance of the ESC 0/1-h algorithm [34, 35].

### Study limitations

First, this survey used a standardized questionnaire to explore clinical practice patterns and information on guideline adherence. The replies reflect the subjective expectations of the lead physician responsible for each CPU, without provision of statistics or objective findings on clinical reality. As such, this survey is not a substitute for a national registry for quality assessment, guideline adherence, or benchmarking. Second, the reported clinical practice patterns indicate a high level of quality of care and very high adherence to the 2015 ESC guideline recommendations. However, the practice pattern was reported from certified CPUs that had passed audits in the past, and involved CPUs affiliated to PCI centers. Thus, a selection bias cannot be excluded. Hence, findings from this survey should be interpreted cautiously and cannot be generalized to other emergency departments or to CPUs without certification from the DGK. Third, physicians were not aware of the 2020 ESC guidelines on NSTE-ACS at the time of the survey. Therefore, physicians had no opportunity to adapt their clinical practice standards for adherence with the updated guidelines. If interpreted in a negative sense, instead of anticipating innovation and state-of-the-art medicine, one could also claim that many centers did not follow the guidelines that were valid at the time of survey.

### Expectations from the survey data

Although we are aware of the fact that data from the current survey originate from intentional ideas rather than given scientific evidence, we strongly interpret our results as hypothesis-generating and best-practice stimulating for those certified CPUs discovering certain gaps in their daily routine as compared to general practice in other certified CPUs. Thus, in addition to its scientific character (with all the limitations of a survey), we also anticipate our data pool to have an educational character. Furthermore, the data originating from our survey should encourage periodic and obligatory patient data collection from all (or representative) certified CPUs on a regular basis addressing benchmarking and quality control. Obligation by the DGK and short-term exemplary data collection similar to the former German CPU registry might be a possible option.

## Conclusion

Anticipating that the survey not only reflects a theoretical intention but also what is done in real life, our survey reaching three quarters of certified German chest pain units (CPUs) supports previous exemplary data from the German CPU registry, indicating an overall high standard of care in those units. With its periodic updates, the German Cardiac Society (DGK) ensures state-of-the-art management in chest pain patients with a special focus on patients with non-ST-segment elevation acute coronary syndrome (NSTE-ACS) or chest pain patients with symptoms of an ischemic origin. The current standard of care is already fulfilling decisive quality criteria of the latest European Society of Cardiology NSTE-ACS guideline, especially for the management of troponin-positive individuals and antiplatelet strategies. Nonetheless, main adaptions will have to affect shorter troponin protocols as well as a more profound role of noninvasive imaging and functional stress testing. In this respect, the usage of treadmill electrocardiogram (ECG) or conventional ergometry as default stress testing at least in community hospitals is a clear shortcoming, Thus, simultaneously, our data underline the importance of regular central benchmarking—a quality measure that should be reintegrated into the oncoming DGK CPU certification criteria to ensure adequate gap analysis and central steering.

## Declarations

For this article no studies with human participants or animals were performed by any of the authors. All studies performed were in accordance with the ethical standards indicated in each case.
